# Women in Surgery: Changing Perspectives

**DOI:** 10.31729/jnma.4124

**Published:** 2019-12-31

**Authors:** Badri Man Shrestha

**Affiliations:** 1 Sheffield Kidney Institute, Sheffield Teaching Hospitals NHS Trust, Sheffield, United Kingdom

Eighth of March is celebrated globally every year as the International Women's Day for what women have achieved and action for gender equality. “Balance for Better” is the theme for the year 2019. The Royal College of Surgeons (RCS) of England celebrates this occasion annually to commemorate the successes and works of many inspirational female role model surgeons of the United Kingdom and implement strategies to encourage and support women to pursue surgical career.^1^ The number of Women in Surgical Training (WIST) and women pursuing long-term surgical career vary between different countries. Despite the recommendation of the Ottawa Consensus of 2010 to the specialty training programmes to aim for producing a workforce that is broadly representative of the population they serve, women remain under-represented in surgical profession.^2^ This editorial summarises the perspectives of Women in Surgery (WinS), particularly, factors those influence women's choice of surgical career and the initiatives those are currently in place to reduce attrition and retain women in surgical profession.

The history of women in surgery dates back to 5000 year ago (3500 before common era), when flints and bronze surgical instruments were discovered from the grave of Queen Shubad of Ur in Mesopotamia. In 19^th^ century, Dr James Barry (1795-1865), a trauma surgeon educated in Edinburgh Medical School, who concealed her sex and practised surgery, was found to be female until his death in 1865. Surgical training has evolved over the years and has attracted WinS because of the perception of possession of “the surgical personality”, successful female and male role models, and the gratification of being able to cope with the intellectual and technical challenges.

Adoption of surgical career by women have been looked upon with scepticism because of the perception of surgery being a male-dominated specialty, demanding nature of the surgical training and the onerous commitments of a consultant needed to deliver high-quality care to the patients, which can be challenging and adversely impact on long-term continuation of surgical career. The reason why women leave surgical training are complex and context dependent. A recently published qualitative study, under the auspices of the Royal Australasian College of Surgeons (RACS) and RACS Training Association, involving 12 WIST who had left the surgical training scheme, identified factors influencing their attrition. These included, inability to take leave, poor mental health, lack of supports from WinS section of their professional body and fear of repercussion if complaints were made.^3^

Similarly, a national survey of the US general surgery residents has evaluated the attitudes, experiences and expectations, which was observed to vary by sex and their training years. Women trainees were more likely to have considered leaving residency due to stress, lack of confidence to perform procedure at the end of their training and lack of support from the system. The postgraduate year 2 and 3 (of 6 years of surgical residency in USA) are difficult times during the training trajectory, as many of the trainees are preparing to complete research years and express worries about their ability to perform procedures and maintain patient safety.^4^ A systematic review and metaanalysis published from USA showed significantly higher attrition rate from surgical residency among females (25% vs. 15%) and identified insufficient role models, lack of institutional support, gender discrimination and harassment, sleep deprivation, adverse interaction with seniors, pregnancies and childbirth and child rearing duties as the causes for attrition.^5^

A recent survey by the WinS working group of the Association of Surgeons of the Great Britain and Ireland has reported that 88% of the female participants perceived surgery as male-dominated field; 59% had experienced discrimination; 58% reported use of gendered language at work place, and poor work-life balance was the main perceived barrier for women in their surgical careers. Fifty percent of the responders perceived barrier to women wanting to pursue and persist with a career in surgery was issues with motherhood and child care commitments.^6^ A survey of final year medical students done in the UK found that the single most important reason for not entering a surgical career was that “it did not fit with lifestyle or family commitments.” This was particularly a problem for female trainees, 85% of whom cited this reason compared with 12% of men.^7^

The WinS, a national initiative of the RCS England committed to inspiring WIST, has been successful in increasing number of women surgical consultants in the UK from 3% in 1991 to 12.2 % in 2018.^8^ In the UK, the proportion of female consultants within surgical specialties include paediatric (27%), plastic (20%), general (gastrointestinal) (16%), faciomaxillary (16%), ear, nose and throat (16%), neurosurgery (7%), vascular (13%), cardiothoracic (8%), urology (10%) and trauma and orthopaedics (7%). The RCS does support women surgeons who wish to work on flexible basis to allow them to achieve an alternative work-life balance. A flexible training and working advisor is appointed by the RCS for this purpose.^9^

A recent news from Nepal highlighting the leadership role taken by Dr. Paleshwan Lakhey as the first women gastrointestinal and general surgical consultant and the head of department at the Institute of Medicine in Kathmandu, is likely to set herself as a role model to encourage women doctors in Nepal to pursue career in surgery.^10^

The solution to the problem of attrition and retention of WIS long-term is to address the issues identified by studies discussed above, provide support to develop resilience and coping mechanisms, maintain professional competency and work-life balance and help develop role models. Providing insights of the nature of the specialty, training scheme, long-term commitments and impact on personal life, starting at the undergraduate medical school level, is important so that motivated candidates can make their choice, which would limit attrition and the loss of surgical training places. It is important to establish a body that would review the problems faced by the WinS regularly, respond to the pressures they encounter and make the scheme attractive and conducive for their retention, which is the way forward in increasing the number of WinS in the coming decades.

## Conflict of Interest

**None.**
